# Medicine, Pathology and Therapeutics

**Published:** 1898-11

**Authors:** Albert T. Lytle

**Affiliations:** Buffalo, N. Y.


### Progress in Medical Science.

### MEDICINE, PATHOLOGY AND THERAPEUTICS.

Conducted by ALBERT T. LYTLE, M. D., Buffalo, N. Y.

### MEASLES IN THE NEW-BORN.

DOUGLAS (*British Medical Journal*, May 7, 1898,) reports a case of measles in the new-born. Ten days after birth, child began to sneeze and show evident indisposition. Three days later, or thirteen days after delivery, child showed well-developed eruptions of measles, covering the body, the eyes congested and the eyelids swollen, the tongue covered with thick fur. There was but little cough and the usual temperature range occurred. This case demonstrates the time of incubation to be ten days, as claimed by Henoch and Osler.

### SYPHILIS OF THE STOMACH.

At the meeting of the Academy of Medicine, held May 17, 1898, M. Dieulafoy discussed the syphilitic affections of the stomach. His conclusions may be summed up as follows: Syphilis of the stomach is not so uncommon as is generally supposed. Syphilitic lesions of the stomach can and do take various forms, from hemorrhagic erosion to gummatous ulceration. Probably the action of gastric juice is a factor in continuing the ulcerative process set up by the syphilis. The symptoms of syphilitic ulceration differ in no way from those of simple ulceration, so that the practitioner should always be on his guard and carefully interrogate any patient suffering from gastric ulcer as to the presence or the reverse of a syphilitic history. Treatment should be begun at once, for in this way a patient may often be relieved by medical measures in whom otherwise it would appear necessary to perform a surgical operation. —*Lancet*, May 28, 1898.

### CREASOTE IN TUBERCULOSIS.

HECTOR MACKENZIE (*Practitioner*, June, 1898,) sums up creasote in the treatment of tuberculosis as follows: While few are probably prepared to admit that creasote has a specific action, most of those who have had a large experience of the drug will admit that it has very valuable properties in the treatment of tuberculosis. The purest beech creasote should be employed. It should at first be given, preferably in the form of capsules, in doses of 1 to 5 minims, three times a day. The dose may be increased to 10 or 15 minims. If under its administration the appetite comes back, the cough and the expectoration diminish, the fever abates, the night sweats cease, strength returns and nutrition improves, the object with which the drug has been given will be attained and happily these are the effects which are often observed. The remedy should be taken after food.

---

### INTUSSUSCEPTION IN CHILDREN.

PACKARD, (*Therapeutic Gazette*, March, 1898,) in a carefully prepared paper, sums up the position of the physician in cases of acute intussusception in young children as follows: "I do believe that injections (to effect reduction by distention of the bowel) are dangerous and oftentimes useless after the third day; that before that date, unless otherwise contraindicated, it is proper to attempt reduction by the use of one thoroughly executed injection of normal salt solution, upon failure of which surgical aid should be at once summoned without loss of time from further attempts by a procedure that should be thoroughly and conscientiously applied in the first instance." . . . "The height of the ceiling of an ordinary room is more than enough to allow of the reservoir of a fountain syringe being elevated sufficiently to rupture the healthy bowel, to say nothing of an intestine seriously damaged by disease."

---

### BEEF TEA AS A FOOD.

PAGE, (*New York Medical Times*,) writing of beef tea as a food, says: "When made in the most approved manner, beef tea is composed of about ninety-seven and one-half parts of water and two and one-half parts of the waste and effete matters of some dead creature. It has, without doubt, some nutritive value; but this is so slight in amount, and its toxic elements so mischievous, that it is doubtful if life would be as long sustained on beef tea as on pure water."

### IODOFORM INTOXICATION.

SASSE is quoted by *Jour. des Practiciens*, October 16, 1897, as recommending the following means of demonstrating in time a threatened iodoform intoxication, a condition which is not rare in surgical and gynecological practice. A test is made of the urine to note the quantity of iodine which is eliminated by it. A small pinch of powdered calomel is placed upon a white saucer, and then a few drops of the urine to be examined are dropped upon it; a mixture of the urine and calomel is then made with a glass rod. If the urine contains a notable amount of iodine there is produced a well-marked yellow discoloration, which should indicate that the iodoform is being absorbed in sufficient quantity to produce danger.—*Therapeutic Gazette.*

---

### INFANT CONSTIPATION.

THOMAS C. MARTIN, (*Medical Science*) after a study of infant defecation and constipation, concludes that difficult evacuation is due from an anatomical standpoint to imperfect development of (*a*) "the expulsory muscularity"; (*b*) to "infant angulation and mobility of the lower intestine, which conspire to obstruct the passage of formed feces"; (*c*) to the "resistance of the rectal valve, which is disproportioned to the expulsory muscularity," and (*d*) to the "insufficient expansibility of the anus, owing to contracted bony pelvic outlet." In consequence, development often relieves the multitudinous consequent ills arising from infantile constipation, retention of feces, straining, prolapsus and ruptures. It would seem that "solution of the stool" is thus the only solution of the problem.

### TANNIGEN.

TANNIGEN is an odorless and tasteless form of tannin, chemically acetyl tannin, insoluble in water and acids, but readily soluble in alkaline solutions—is quite a valuable remedy in diarrhea, chiefly in children. Its continuous use is apparently without disagreeable effects. Dr. J. Vandenberghe reports that the administration of this agent, accompanied with proper feeding, saved many children in his practice who were suffering from various forms of acute and chronic enteritis; improvement was marked, rapid and resulted in complete recovery. Dr. J. Conley, after a prolonged trial in the treatment of infantile diarrhea, asserts satisfactory results in simple noninfective forms in children, but that it is not adapted to cholera infantum, unless some antiseptic, like calomel, is given with it.—EPHEMERIS.

### ANASIN.

ANASIN is a new synthetic, hypnotic and anesthetic, introduced by manufacturers in Basle, Switzerland. It is claimed to be an aqueous solution of tri-chlor-pseudo-butyl-alcohol, or aceto-chloroform. It is said to resemble chloral hydrate in its hypnotic action, the dose being 500 mgs. (7.7 grains) to 1 gramme (15.4 grains). Even the maximum dose may be administered without producing ill effects. A 1 per cent. solution is found to have the same anesthetic properties as a 2 to 2½ per cent. solution of cocaine hydrochlorate. Its advantages are freedom from toxicity, nonirritability when applied locally, keeping well and possible sterilisation. It has been used in the eye, injected subcutaneously, applied to the larynx, pharynx and nasal mucous membrane and in dental operations. When applied to the tongue or eyes, for instance, the anesthesia is slow in developing, as its diffusibility is low, thus going to show that it must in all cases be applied directly to the spot requiring its anesthetic action. It has an advantage over cocaine as used in the eye, in that it does not produce mydriasis. The iris is not affected by it.—EPHEMERIS.

### SALOL.

Two cases of calculi formed during the long administration and rather large dosage of this drug have been reported. The mode of giving was in cachet; a careful study of the cases indicates that salol is but slowly decomposed in the alimentary tract, and may give rise to symptoms of obstruction. This may be obviated to some extent by rubbing it up with some innocuous powder.

### FOR AMYGDALITIS AND NONDIPHTHERITIC ANGINAS.

| | | |
|---|---|---|
| ℞ | Salol | 2.00 gms. |
| | Ol. amygdalæ dulcis | 4.00 cc. |
| | Syr. simplicis | 30.00 cc. |
| | Aquæ destil | 75.00 cc. |

Sig.—To be taken in three doses during the day.

—EPHEMERIS.

### FOR MIGRAINE.

| | | |
|---|---|---|
| ℞ | Methylene blue | 1.50 gms. (23 grains.) |
| | Pulv. Myristiæ | 1.50 " (23 " ) |

M. et div. in capsulæ No. 15.

Sig.—One capsule, three or four times a day.

—LEWY.

FOR PHTHISIS IN CHILDREN.

| | | |
|---|---|---|
| ℞ | Beechwood creasote | 3.00 gms. to 8.00 gms. |
| | Ol. gaultheriæ | 0.60 " |
| | Pulv. acaciæ | 3.00 " |
| | Glycerini | 15.00 " |
| | Ol. morrhuæ q. s. ad | 175.00 " |

M. et. Sig.—One teaspoonful one hour after each meal.

—EPHEMERIS.

FOR BLEEDING MUCOUS SURFACES (EPISTAXIS).

| | | |
|---|---|---|
| ℞ | Antipyrin | 0.50 gms. |
| | Tannin | 1.00 " |
| | Powd. sugar | 10.00 " |

M. et Sig.—Dust a little on bleeding surface several times a day.

—RENDU.

APHTHOUS STOMATITIS.

| | | | |
|---|---|---|---|
| ℞ | Sodii biboratæ | ʒi. or | 4.00 |
| | Tinct. benzoin | ʒss. " | 2 |
| | Aquæ destil. | ℥iii. " | 15.00 |
| | Syrup simplicis | ℥vi. " | 30.00 |

M. et Sig.—Use as mouth wash five or six times a day.

—*Jnl. des Pract.*

TO MASK TASTE OF QUININE.

EPHEMERIS advises the syrup of rose leaves, upon the suggestion of Mr. E. N. Gates, as an excipient to mask the taste of quinine sulphate. The mixture must not be diluted with water or the taste of the quinine becomes evident, otherwise only the peculiar bitter taste of rose leaves is noticeable. The syrup is made from three parts simple syrup and one part fluid extract rosæ centifolia. The proportion of quinine to syrup is, maximum drug, ten grains, to syrup one fluid drachm.

PROTARGOL IN OPHTHALMIA NEONATORUM.

CHENEY (*Boston Med. and Surg. Jour.*, August 25, 1898,) says that he has used this drug in 130 patients in the Massachusetts Charitable Eye and Ear Infirmary and that it seems to possess all the advantages of nitrate of silver, with none of its disadvantages. The very slight degree of irritation produced, and the nearly complete absence of pain, are its chief claims to trial. A 2 or 4 per cent. solution can be used without cocaine, and it can be applied with a brush, with absorbent cotton on a probe, or with a dropper. Dr. Cheney regards it as equally efficient and much less disturbing than silver nitrate.

### DIABETES MELLITUS TREATED WITH BENZOSOL.

N. B. ASPINWALL (*American Therapist*) reports three cases of the disease treated with benzosol with favorable results :

CASE I.—Man, 48 years old, suffering from polyuria, frequent urination, thirst, progressive emaciation, dryness of skin, a general pruritus; in short, the usual symptoms of diabetes mellitus. After other remedies had been tried the patient was given five grains of benzosol every four hours. Under this treatment the patient rapidly improved in general health and increased steadily in weight. The urine decreased in quantity, the specific gravity gradually receding to 1.020, and the sugar decreased progressively until it entirely disappeared.

CASE II.—Mr. E. B. Similar in all respects to case above related, except that the benzosol treatment was commenced as soon as the patient came under Dr. Aspinwall's care. The results were equally prompt and satisfactory, and at the date of the report the patient passed a normal amount of urine of a specific gravity of 1.020.

CASE III.—Mrs. G.; widow; 50 years; has been under treatment for two months for the same disease. Benzosol, five grains, four times a day, is bringing about rapid improvement.

### PEPTO-MANGAN (GUDE) IN SURGICAL CONVALESCENCE.

STUART MCGUINE (*Virginia Med. Semi-monthly*) reports 20 patients treated with this remedy, giving the blood count in them all. The following is selected as a specimen of the cases and as showing a good test:

CASE V.—Miss E. J., 17 years old. Patient St. Luke's hospital. Spinal irritation from a fall. Anemic, emaciated and confined to bed for more than a year from contraction of ham-string muscles. Electricity, massage and passive movements. Gave Gude's pepto-mangan forty days. First count, 3,650,000 red corpuscles to the cubic millimeter; second count, 4,425,000 to the cubic millimeter. Her menses, which had been suppressed, became regular. She fattened twenty pounds and left the hospital walking with a cane.

In all, the blood counts showed improvement under the use of the remedy.

